# Best zero level for external ICP transducer

**DOI:** 10.1007/s00701-019-03856-x

**Published:** 2019-03-08

**Authors:** Peter Reinstrup, Mårten Unnerbäck, Niklas Marklund, Wilhelm Schalen, Jesus Cabrera Arrocha, Eric L. Bloomfield, Vahabi Sadegh, Karin Hesselgard

**Affiliations:** 1 Department of Intensive & Perioperative care, Skanes University Hospital, Lund, Sweden; 2 Department of Intensive & Perioperative Care, Skanes University Hospital, Malmö, Sweden; 3 Department of Clinical Sciences, Neurosurgery, Skane University Hospital, Lund, Sweden; 4 Department of Intensive Care, Gran Canaria University Hospital, Las Palmas, Spain; 5 Department of Anesthesiology/CCM, Mayo Clinic, Rochester, MN USA; 6 Department of Neuro Radiology, Skanes University Hospital, Lund, Sweden

**Keywords:** Intracranial pressure, ICP, Zero reference point, Monitoring

## Abstract

**Background:**

Continuous monitoring of intracranial pressure (ICP) was introduced in the 1950s. For correct ICP recordings, the zero-reference point for the external pressure gauge must be placed next to a head anatomical structure. We evaluated different anatomical points as zero reference for the ICP device at different head positions and their relation to brain centre (BC), foramen of Monro (Monro), and brain surface.

**Methods:**

Patients referred for neuroimaging due to e.g. headache all having normal 3D MRI scans were selected. Monro, BC, Orbit(O), external auditory meatus (EAM), and orbito-meatal (OM) line were identified and projected to mid-sagittal, or axial images. Each scan was evaluated like lying supine, 45° head elevations, upright, and 45° lateral position. Distances from skin to brain surface, BC, and Monro were measured. All values are presented as mean ± SD and/or range in millimetre. For conversion to mmHg, millimetre was multiplied by 0.074.

**Results:**

Twenty MRI scans were examined. A zero reference at EAM or glabella was ideal at BC when head was strict supine or in the lateral position. At 45° head elevation, an overestimation of the BC-ICP by 4.8 ± 0.8 and in upright 5.6 ± 0.5 mmHg was found, and 45° lateral underestimated ICP-BC by 6.3 ± 1.0 mmHg. Monro was situated 45 ± 5 mm rostral to the mid-OM line and 24 (18–31) mm inferior and 13 (8–17) mm in front of BC. A zero-reference point aligned with the highest point of the head underestimated BC-ICP and Monro-ICP. If the ICP reading was added 5.9 or 6.3 mmHg, respectively, a deviation from BC-ICP was ≤ 1.8 mmHg and Monro-ICP was ≤ 0.9 mmHg in all head positions.

**Conclusions:**

EAM and glabella are defined anatomical structures representing BC when strict supine or lateral but with 12 mmHg variation with different head positions used in clinical practice. The OM line follows Monro at head elevation, but not when the head is turned. When the highest external point on the head is used, ICP values at brain surface as well as Monro and BC are underestimated. This underestimation is fairly constant and, when corrected for, provides the most exact ICP reading.

## Introduction

Intracranial pressure (ICP) monitoring is commonly performed using an intraventricular catheter connected through a fluid-filled system to an external pressure gauge. Such a system can be calibrated regularly to the atmospheric air pressure to avoid drift and is commonly regarded as the “gold standard” [1]. Although the system provides highly accurate measurements of pressure changes, the absolute values are dependent on the chosen reference point on which the gauge is placed.

With regard to the reference point, no defined “gold standard” exists. The pressure transducer is placed outside the head and the gauge level needs to be related to an anatomical structure on the head surface. This can be done while placing it at brain midpoint, Origo, or brain centre (BC), at the centre between the foramen of Monro (Monro), or at the surface of the brain.

In available literature, a variety of different external anatomical land marks have been used [2–4] and none of which have been evaluated. When the patient is in supine position, common transducer placements are the external auditory meatus (EAM), at the orbito-meatal (OM) line (Orbit: outer cantus of the eye), midpoint between the Orbit and top of the outer ear, 2 cm above the mid-OM line, and at the highest point on the head. When the patient is in the lateral position, the commonly used reference points are the nose tip or glabella (midline between the eyebrows) and the highest point of the head.

It is important to register a correct ICP both for the ICP value itself and also for the calculated CPP value. With this study, we wanted to evaluate some of the commonly used placements for the zero-reference point of the ICP device and correlate their relation to the BC, Monro, or brain surface at clinically used positions of the head by evaluating 3D MRI scans.

## Methods and material

Images from 20 consecutive patients (10 females and 10 males, aged 20 to 60 years) admitted to MRI scanning at the Neuroradiological Department due to non-specific diagnoses such as headache were included. All MRI scans, 3D T1, T2, or Flair, space, or MP-RAGE with 124 to 196 pictures were identified in our x-ray handling system (Sectra AB, Linköping, Sweden). The pictures were only included in our study if they had an intact cranial cavity, no space-occupying lesions, and with apparent normal age-calibrated cerebrospinal fluid spaces. Age was noted and then the pictures were unidentified.

**Fig. 1:**
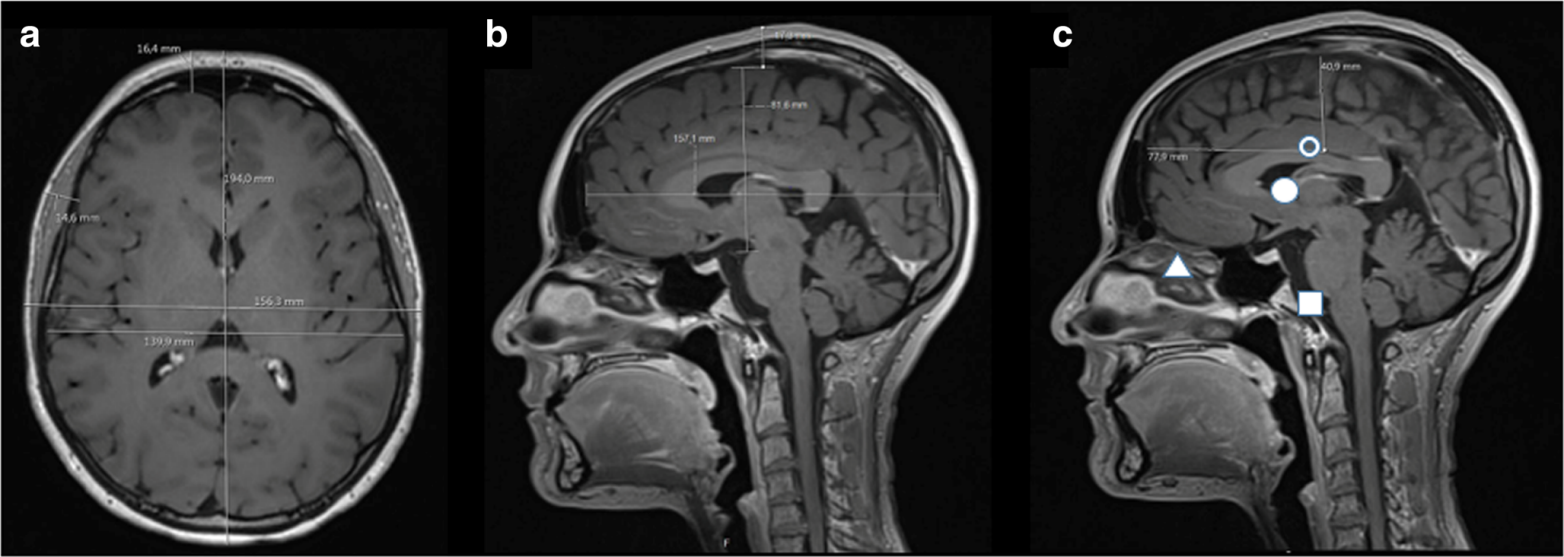
Basic measurement. **a** Axial image where the head length and width as well as brain width along with the skin to brain surface were measured. **b** Mid-sagittal picture where the brain length and height were measured. **c** Mid-sagittal picture with brain centre (BC) (clear circle), meatus (white square), Monro (white circle), and Orbit (cantus) (white triangle)

The length and width of the head and brain were measured (Fig. 1a, b). The supratentorial height of the brain was defined as the distance from the posterior part of the sella turcica level to the highest point around vertex (Fig. 1b). The BC of the brain was defined on the sagittal picture by taking half the length and height of the brain (Fig. 1c). One foramen of Monro was identified on a lateral sagittal image and transferred to the midline sagittal (Monro) (Fig. 1c). The Orbit, defined as the cantus, was identified on a lateral sagittal image and projected to the midline sagittal (Fig. 1c). The meatus was identified and transferred to the midline sagittal picture (Fig. 1c).

**Fig. 2:**
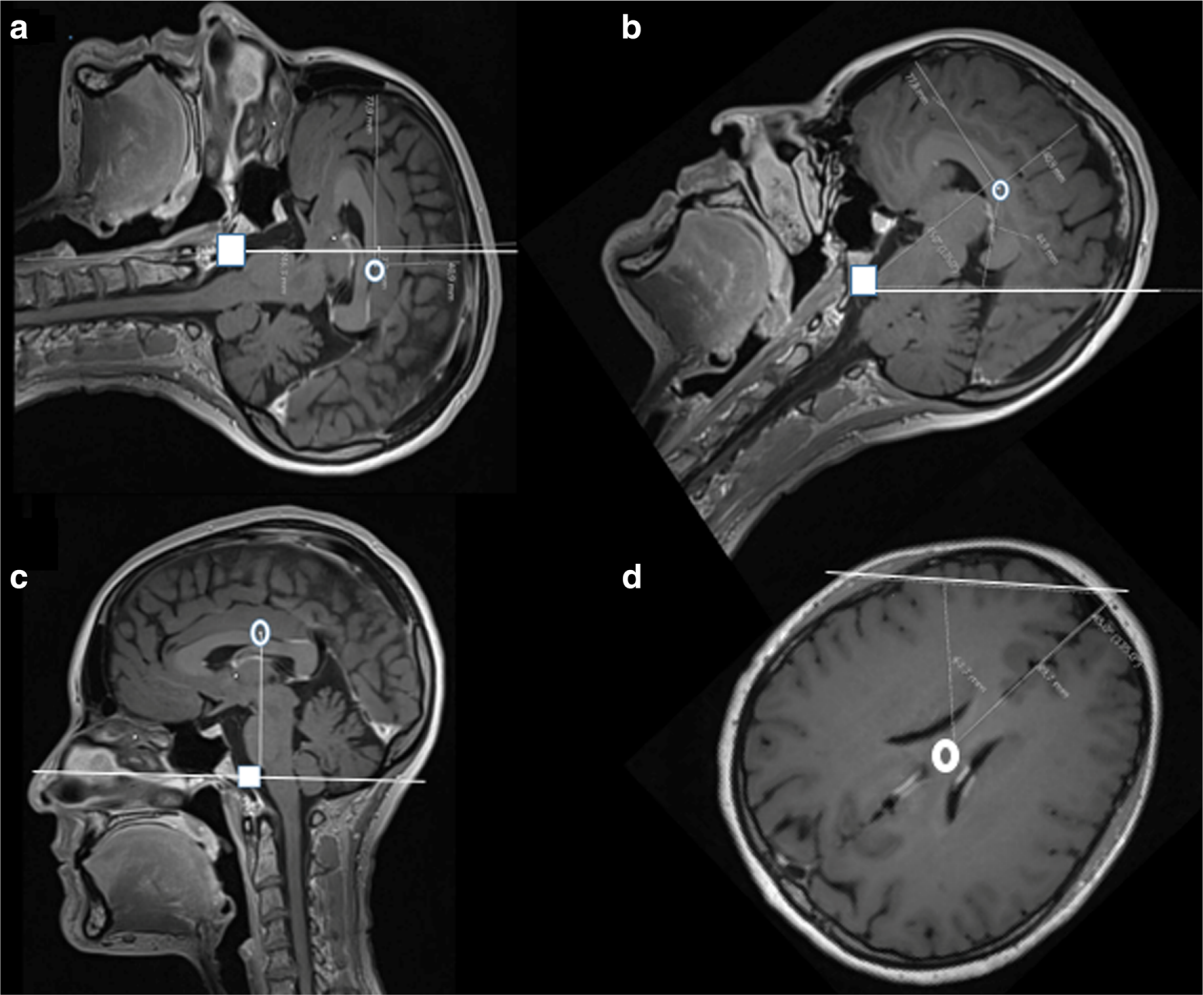
White line represents placement of the ICP gauge at external auditory meatus (brain centre (BC) (clear circle); meatus (white square)). **a** Sagittal image with the head in supine position. **b** 45° head elevation. **c** Upright. **d** White line at glabella-nose tip with placement in 45° lateral position

The meatus and glabella are assumed to represent BC at different head positions. With the meatus projected to a mid-sagittal on an axial slice, the deviation from BC could be evaluated when the scans were with patients lying supine (Fig. 2a), supine with 45° head elevations (Fig. 2b), and upright (Fig. 2c). If the patient was lying in the lateral position, the glabella was used as reference point with the deviation from the BC being measured when the head was turned 45° (Fig. 2d).

**Fig. 3:**
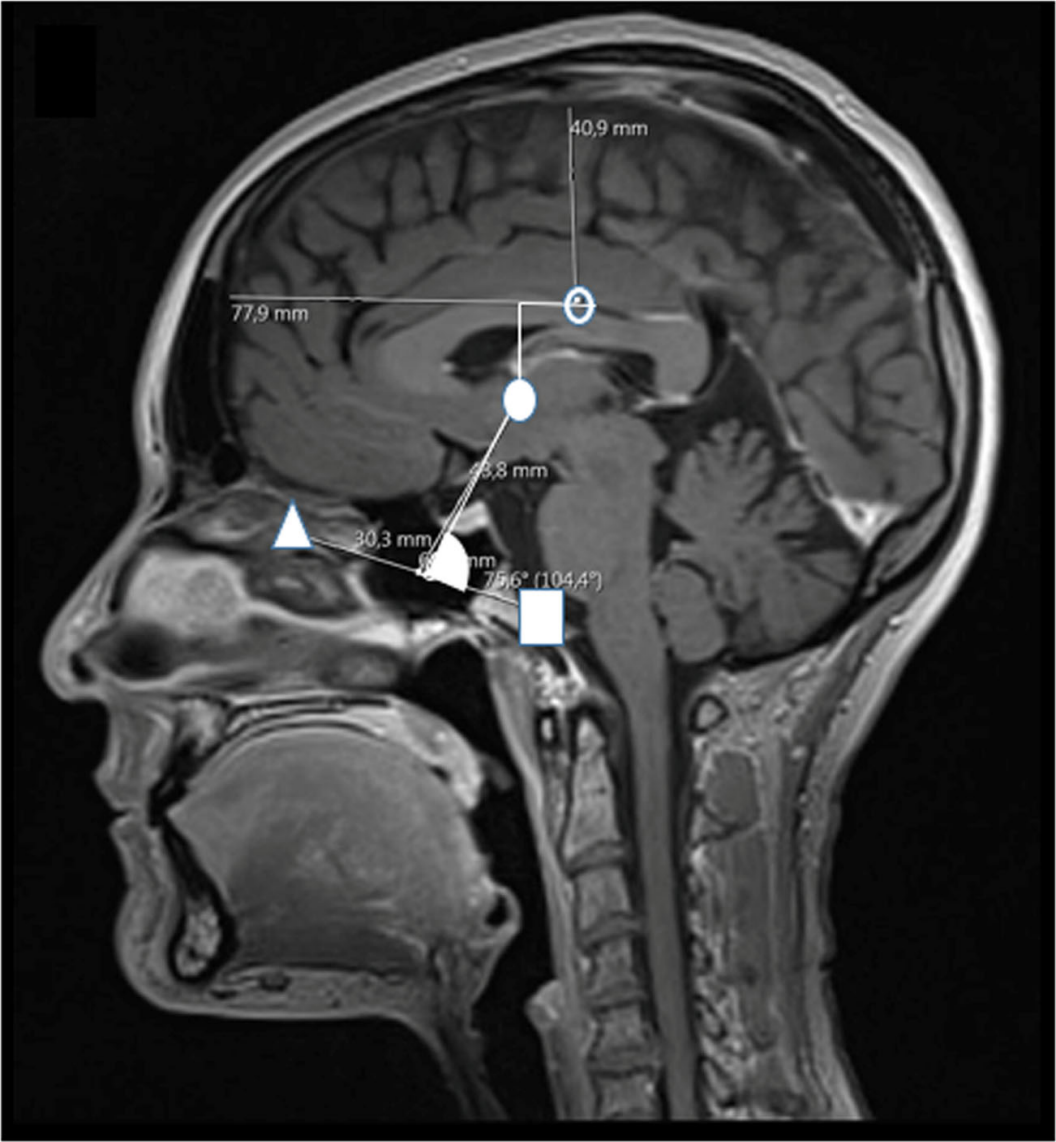
Sagittal picture with brain centre (BC) (clear circle), meatus (white square), Monroe (filled circle), Orbit (cantus) (white triangle), and the measured distances and angle

The distance from BC to Monro in the horizontal and vertical plane was measured on the sagittal images (Fig. 3). In addition, we measured the distance from the midpoint of the OM line to Monro and the angle from meatus—midpoint of the OM line to Monro (Fig. 3).

**Fig. 4:**
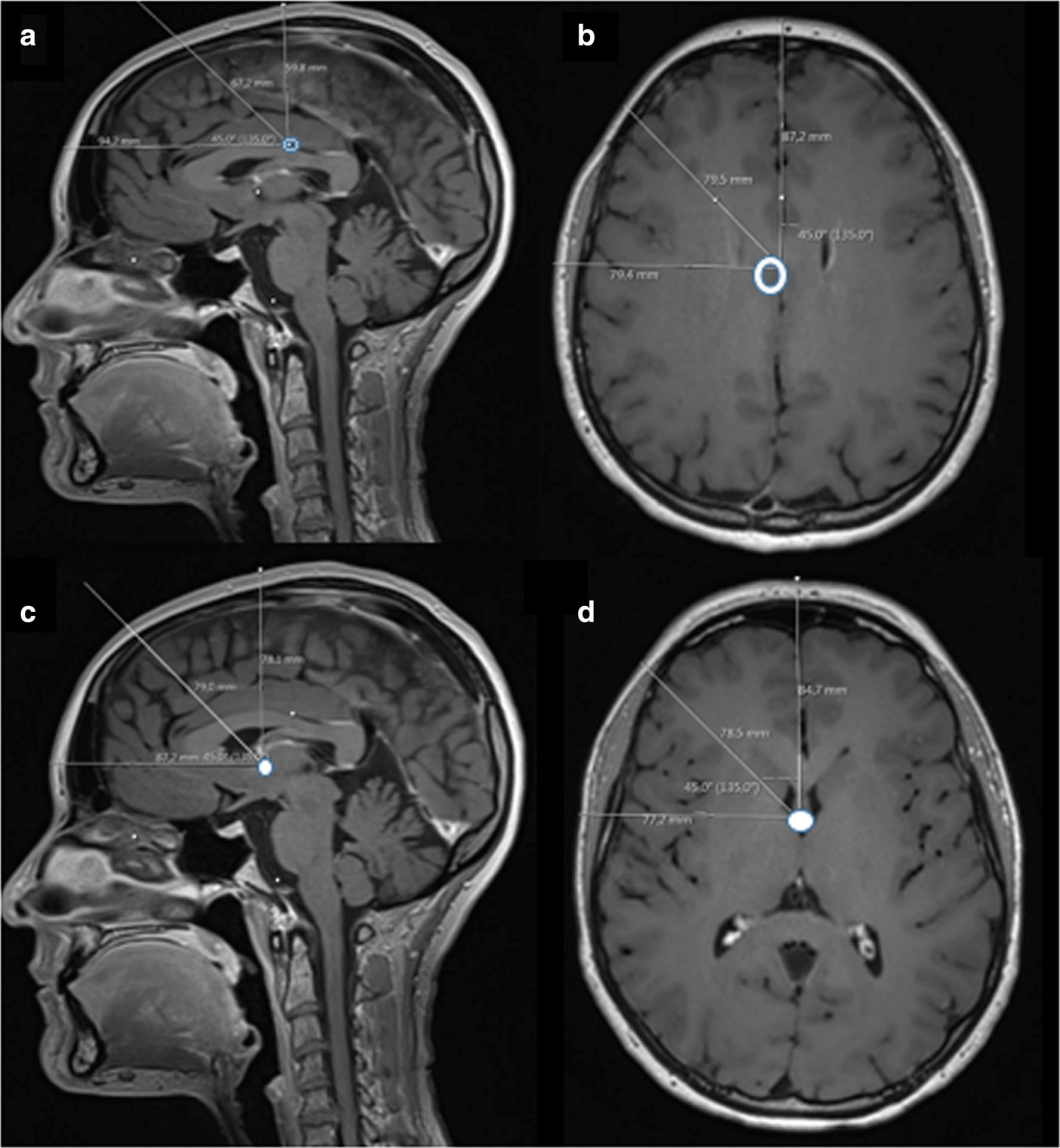
Sagittal and axial pictures with the distances from the highest external point of the head to brain centre (BC) (clear circle) (**a**, **b**) and Monro (white circle) (**c**, **d**) using the highest point of the head at supine, upright, 45° head elevation, strict lateral, and 45° lateral

Furthermore, the distance while in the supine position, 45° head lift, and upright position on sagittal images from the skin to both BC (Fig. 4a) and Monro (Fig. 4c) was determined. On axial images, we measured the distances from skin to both BC (Fig. 4b) and Monro (Fig. 4c) in strict lateral, 45° lateral, and supine position.

All values are presented as mean ± SD and/or range unless stated otherwise. All values are converted from the distance in millimetre to a measurement of pressure (mmHg) when appropriate by multiplying by 0.074 as the connection between the ventricular catheter to the external pressure gauge is through a saline filled system.

## Results

In patients, a zero reference point at EAM was − 5 ± 6.4 (− 14–10) mm below BC or deviated from BC-ICP by 0.4 ± 0.5 (− 1–0.7) mmHg when strict supine (Fig. 2a, Table 1 (9), Table 2 (9)). With the head angled 45° from the horizontal plane, the EAM distance to BC decreased to 65 ± 11 (43–86) mm and overestimated the BC-ICP pressure by 4.8 ± 0.8 (3.2–6.3) mmHg (Fig. 2b, Table 1 (10), Table 2 (10)). Sitting position EAM to BC was 76 ± 6 (66–87) mm under or 5.6 ± 0.5 (4.9–6.4) mmHg higher than ideal BC-ICP (Fig. 2c, Table 1 (11), Table 2 (11)). With the head at the side but turned 45°, the transducer would aim 85 ± 13 (57–116) mm above BC and as a result, the ICP figure would underestimate the BC-ICP by 6.3 ± 1.0 (4.2–8.6) mmHg (Fig. 2d, Table 1 (12), Table 2 (12)).

**Table 1:** The different measured distances and angles are described in the left column. The italicized number and letters refer to the images in the different figures where the measurement was obtained. *OM* orbito-meatal line. *BC* brain centre: half the brain length and height point. All values except for angles are given in millimetre

	Female			Male		
	Mean	SD	Range	Mean	SD	Range
Age (years)	40	10	26–53	42	9	27–60
Basic measurements in mm						
1. Head length *1a*	191	5	186–200	205	11	154–221
2. Head width *1a*	150	6	140–159	162	5	154–169
3. Brain length *1 a+b*	159	4	153–165	165	10	151–179
4. Brain width *1 a*	124	8	108–136	128	5	116–136
5. Brain height *1 b*	83	4	78–89	87	5	79–97
6. Skin-brain surface front *1 a*	14	2	11–20	15	2	11–19
7. Skin-brain surface vertex *1 b*	17	3	13–22	17	2	15–19
8. Skin-brain surface temporal *1A a*	15	1	14–16	18	2	14–21
BC measurements						
9. Meatus-BC supine *2 a*	− 6	6	− 14–7	− 4	7	− 12–10
10. Meatus-BC 45° up *2 b*	47	5	39–54	51	6	40–58
11. Meatus-BC sitting *2 c*	74	6	67–84	78	6	66–87
12. Glabella-BC 45° turned midbrain *2 d*	67	2	64–70	69	5	62–76
BC to Monro						
13. BC-Monroe inferior *3*	23	4	18–31	25	3	18–30
14. BC-Monroe frontal *3*	13	3	8–17	13	2	11–18
OM line to Monro						
15. OM to Monroe *3*	45	6	39–56	46	4	41–53
16. OM mid to Monroe angle in ° *3*	77	8	67–95	80	7	73–97
Skin BC measurements						
17. Skin BC front *4 a*	93	3	90–99	97	4	93–104
18. Skin BC 45° up *4 a*	70	3	66–75	73	3	69–80
19. Skin BC vertex *4 a*	60	3	56–66	60	3	55–67
20. Skin BC temporal *4b*	76	6	70–90	79	3	75–84
21. Skin BC side 45° *4B b*	82	8	60–87	81	14	59–94
Skin-Monro measurements						
22. Skin-Monro frontal *4 c*	85	2	82–88	91	4	84–98
23. Skin-Monro 45° up *4 c*	83	4	77–87	87	5	80–96
24. Skin-Monro vertex *4 c*	82	5	74–87	84	5	77–92
25. Skin-Monro temporal *4 d*	74	2	71–78	79	3	74–84
26. Skin-Monro side 45° *4D d*	79	3	72–82	85	5	78–95

**Table 2:** Deviations from ideal brain centre (BC) in mmHg at different head positions using meatus (9–11) or glabella (12) as anatomical landmarks. 17–21 deviation from BC with the transducer levelled at the highest external point of the head at different positions of the head. 22–26 the same deviation from Monro and the transducer at the highest external point of the head. The italicized number and letters refer to the figures where the measurement took place. BC: half the brain length and height point. All values are given in mmHg with mean, SD, and range

	Female			Male		
	Mean	SD	Range	Mean	SD	Range
Meatus BC measurements						
9. Meatus-BC supine *2 a*	− 0.4	0.4	− 1.0–0.5	− 0.3	0.5	− 0.9–0.7
10. Meatus-BC 45° up *2 b*	3.5	0.4	2.3–4.1	3.8	0.4	3.0–4.3
11. Meatus-BC sitting *2 c*	5.5	0.4	5.0–6.2	5.8	0.4	4.9–6.4
12. Glabella-BC 45° turned *2 d*	5.0	0.2	4.7–5.2	5.1	0.4	4.6–5.6
Skin BC measurements						
17. Skin BC front *4 a*	6.9	0.2	6.7–7.3	7.2	0.3	6.9–7.7
18. Skin BC 45° up *4 a*	5.2	0.2	4.9–5.6	6.1	0.2	5.1–5.9
19. Skin BC vertex *4 a*	4.4	0.2	4.1–4.9	4.4	0.2	4.1–5.0
20. Skin BC temporal *4 b*	5.6	0.4	5.2–6.7	5.8	0.2	5.6–6.2
21. Skin BC side 45° *4 b*	6.1	0.6	4.4–6.4	6.0	1.0	4.4–7.0
Skin-Monro measurements						
22. Skin-Monro frontal *4 c*	6.3	0.2	6.1–6.5	6.7	0.3	6.2–7.3
23. Skin-Monro 45° up *4 c*	6.1	0.3	5.7–6.4	6.4	0.4	5.9–7.1
24. Skin-Monro vertex *4 c*	6.1	0.4	5.5–6.4	6.2	0.4	5.7–6.8
25. Skin-Monro temporal *4 d*	5.5	0.2	5.3–5.8	5.8	0.2	5.5–6.2
26. Skin-Monro side 45° *4 d*	5.8	0.2	5.3–6.1	6.3	0.4	5.8–7.0

Monro was situated 45 ± 5 mm rostral to the mid-OM line. In relation to BC, Monro was situated 24 (18–31) mm inferior and 13 (8–17) mm in front (Fig. 3, Table 1 (13 + 14)).

A zero reference point aligned with the highest point of the head underestimated BC in all patients and positions of the head by 77 ± 13 (55–104) mm or BC-ICP by 5.7 ± 1.0 (4–7.7) mmHg average 5.9 (Fig. 4a, b, Table 1 (17–21), Table 2 (17–21)). When the highest point on the head reading was corrected by adding 5.9 mmHg, the ideal BC-ICP deviated by ≤ 1.8 mmHg.

A zero reference point aligned with the highest point of the head underestimated Monro in all patients and positions of the head by 83 ± 5.7 (71–98) mm or Monro-ICP by 6.1 ± 0.4 (5.3–7.3) mmHg average 6.3 (Fig. 4c, d, Table 1 (22–26), Table 2 (22–26)). When the highest point reading was corrected by adding 6.3 mmHg, the ideal Monro-ICP deviated by ≤ 0.9 mmHg.

In general, the measurements from the images are presented in millimetre in Table 1 and the clinically relevant values converted to mmHg in Table 2.

## Discussion

There are numerous publications describing ICP, the most common monitoring parameter in neurosurgical intensive care practice. However, most of these do not define the zero-reference point [5]. An intraparenchymal pressure device has its zero point at, or close to, the tip of the catheter. In order to evaluate the exact zero point of such a catheter, a brain scan is needed to visualise tip placement and to correlate it to patient position. Most intraparenchymatous catheters are placed in the right frontal lobe, and accordingly, the zero-reference point is at that level. Previous studies compared the correlation between a ventricular catheter with an external gauge and different intraparenchymatous catheters [6–9] and found parenchymatous pressure deviations from the ventricular readings. This can be explained by deviations in the zero-reference point for the different techniques and not necessarily due to drift of the parenchymatous device [8, 9].

The gold standard technique with a ventricular catheter can be recalibrated to atmospheric air both to reduce drift and correct for changes in atmospheric pressure, always providing the correct pressure. However, the zero reference depends on where the external located pressure transducer is placed. It is vital to stress that different centres use different zero reference points [2, 4, 10–12], surprising in view of the clinical importance of CPP calculation.

Modern technology using laser pointers makes the placement of a zero point at different anatomical structures on the head easy although carries a risk of eye damage [13, 14].

To obtain a strict BC zero point, the meatus [2, 10, 12] with the patient supine or the glabella when the patient is in a lateral position are two often used methods. In our study, a zero point at the meatus was found ideal when the patient’s head was placed supine without head elevation. This method resulted in only minor deviations from the BC-ICP, ranging from – 1.0 to + 0.5 mmHg. However, by elevating the bed rest and/or just placing a pillow under the head alters the angle and at 45° position from horizontal overestimates the ICP from the ideal BC position by 4.8 mmHg, and in the upright position, as used in e.g. awake patients with subarachnoid haemorrhage, the overestimation can be as high as 5.6 mmHg. When the head is in a strict lateral position, the glabella is an ideal BC zero point but if the head was turned 45° to the side, an underestimation of 6.3 mmHg was found. In clinical neurocritical routine, a combination of these head positions is common, which makes these anatomical reference points unfavourable if an absolute and reproducible BC-ICP is aimed for. In lack of methods for online CBF measurements, CPP-guided therapies are common for traumatic brain injury. If the meatus-glabella is used as zero reference for the ICP, different positions of the head could influence BC-ICP reading with 12 mmHg. Thus, the Brain Trauma Foundation guidelines [15] for management of severe traumatic brain injury recommended that further improvement in ICP monitoring technology should focus on developing multiparametric ICP devices.

Alternative zero reference points were described in 1999. One was described as 1 cm posterior to the Orbit, halfway between the tragus of the ear and the outer corner of the eye while another was described as an imaginary line between the top of the ear and outer cantus of the eye [3]. It is, however, unclear if and where such procedures are currently in use. A more often described and plausibly better reference point concerning stability is the foramen of Monro [11] which is situated 24 mm (range18–31) inferior and 13 mm (range 8–17) in front of BC. This results in a difference from the ideal BC-ICP related to these measurements and position of the head. The external anatomical points related to Monro have been described at pterion or 20 mm above the pterion [2]. However, the pterion is not an external anatomical structure. Other studies find Monro to be situated at or above the middle of the OM line [16]. In our study, we find the foramen Monro to be 45 mm (range 39–56) superior to the OM lines midpoint with a mean 78° (range 67°–97°) angle from meatus. The previously described mid-OM line as an external anatomical point when used to find the foramen of Monro was therefore not perfect. However, the advantage in using the foramen of Monro technique, if found, is that the patient’s head can be elevated while keeping the transducer at the same brain level. Nevertheless, head turning creates marked difficulties when externally trying to estimate the position of Monro.

In the work of Nils Lundberg nearly 60 years ago [4], the pressure transducer was at all time at the highest external point of the head. This procedure has the advantage of being the most obvious anatomical landmark to position the pressure transducer using a spirit, bubble, or carpenters’ lever in all head positions. This zero-reference point always provide a zero point at the brain surface minus 0.8 to 1.5 mmHg (see Fig. 1a, b).

Concerning the highest point of the head and its relation to the BC, there is a correlation to half the head length when the patient is lying supine, and half the width when the head is lateral. The shortest distance is to the vertex and the longest from BC to the frontal skin with a mean difference of 36 mm (range 25–42). This results in a difference in ICP reading of 2 to 3 mmHg when the head is repositioned from supine to fully upright. In order to adjust the acquired measurements to a BC level, a correction factor has to be applied. The correction factor should equal half the head width, corresponding to “skin midbrain temporal” with a range of 70 to 90 mm. Adding 80 mm (5.9 mmHg) as a fixed correction digit results in a deviation within the range of ≤ 1.8 mmHg in all clinical positions of the head.

The Monro is situated in front and below the BC (Fig. 3) making the skin to Monro distance more equal in all clinical positions of the head. The distance from the highest point of the head to Monro was 71 to 98 mm from the skin. If 6.3 mmHg is added to the highest point of the head ICP reading, the deviation from the foramen of Monro-ICP as the zero-reference point would be ≤ 0.9 mmHg in all clinically relevant positions of the head.

Whatever reference point is used, the level of an external transducer needs to be adjusted with each change in head position. Importantly, the CPP is regarded as independent of the placement of the ICP transducer if the arterial blood pressure (ABP) transducer is placed at the same level as the ICP transducer [12, 17, 18] (or adjusted for the distance between the transducers). However, if for instance the EAM and glabella are used as zero reference points, BC-ICP may deviate from plus 4.8 to minus 6.3 mmHg in clinical practice as will the correct CPP, but not the measured. The policy of transducer positioning differs between centres but the ABP transducer is mainly positioned at the heart level posing a more profound divergence in CPP [5].

To date, there is no consensus about where to place the pressure transducer and how to use any correction factor [5, 19, 20]. A uniform procedure worldwide would be warranted and the highest point of the head with an addition of 6.3 mmHg resulting in a zero at the Monro is the most reliable. Most articles containing information about ICP do not provide information regarding the zero-reference point for the ICP transducer nor the head position [5] or the location of the ABP transducer needed for CPP calculations [5, 19, 20]. In view of the deviations that may occur with the different measurement methods and position of the patient used in the different centres, this information should be mandatory in future reports.

## Conclusion

The meatus and glabella are well-defined anatomical structures but should not be advocated as zero reference points for the ICP gauge in clinical practice. The foramen of Monro is not a well-defined external head structure. If found and used, this reference point represents the Monro-ICP during head elevation but when the head is turned, the foramen of Monro level has to be arbitrarily decided. Both the above zero reference points should therefore be avoided in clinical practice. A pressure transducer in line with the highest external point on the head is easy to use. It underestimates the ICP at brain surface, BC, and Monro, but this underestimation is fairly constant and can easily be corrected for. At present, a corrected ICP levelled at the highest point of the head represents the closest to gold standard for measuring ICP in clinical practice.
